# COVID-19 outcomes in hospitalized Parkinson’s disease patients in two pandemic waves in 2020: a nationwide cross-sectional study from Germany

**DOI:** 10.1186/s42466-022-00192-x

**Published:** 2022-07-11

**Authors:** Raphael Scherbaum, Dirk Bartig, Daniel Richter, Eun Hae Kwon, Siegfried Muhlack, Ralf Gold, Christos Krogias, Lars Tönges

**Affiliations:** 1 Department of Neurology, St. Josef-Hospital, Ruhr-University Bochum, 44791 Bochum, Germany; 2 DRG MARKET, 49069 Osnabrück, Germany; 3 Neurodegeneration Research, Protein Research Unit Ruhr (PURE), Ruhr-University Bochum, 44801 Bochum, Germany

**Keywords:** Parkinson’s disease, COVID-19, Mortality, Intensive care, Health care utilization

## Abstract

**Background:**

The individualized clinical and public health management of the COVID-19 pandemic have changed over time, including care of people with PD. The objective was to investigate whether in-hospital COVID-19 outcomes and hospital care utilization of people with PD differed between the first two pandemic waves (W) 2020 in Germany.

**Methods:**

We conducted a nationwide cross-sectional study of inpatients with confirmed COVID-19 and PD between March 1 and May 31 (W1), and October 1 and December 31 (W2), 2020 and 2019, using an administrative database. Outcomes were in-hospital mortality, ICU admission rate, change in hospital care utilization, demographical data, PD clinical characteristics, and selected comorbidities. Differences were assessed between waves, PD/non-PD groups, and years.

**Results:**

We identified 2600 PD COVID-19 inpatients in W2 who in total showed higher in-hospital mortality rates and lower ICU admission rates, compared to both W1 (n = 775) and W1/W2 non-PD COVID-19 inpatients (n = 144,355). Compared to W1, W2 inpatients were more long-term care-dependent, older, more of female sex, and had less advanced disease. During both waves, PD inpatients were older, more frequently male and long-term care-dependent, and showed more risk comorbidities than non-PD COVID-19 inpatients. Decreases in hospital care utilization were stronger than average for PD inpatients but relatively weaker during W2. Non-COVID-19 PD inpatients showed poorer in-hospital outcomes in 2020 than in 2019 with better outcomes during W2.

**Conclusions:**

In-hospital COVID-19 outcomes and hospital care utilization of PD patients in Germany differed between the two pandemic waves in 2020 with increased in-hospital mortality for PD COVID-19. Overall hospital care utilization for PD was increased during W2.

***Trial registration*:**

No trial registration or ethical approval was required because data were publicly available, anonymized, and complied with the German data protection regulations.

**Supplementary Information:**

The online version contains supplementary material available at 10.1186/s42466-022-00192-x.

## Background

The severe acute respiratory syndrome coronavirus 2 (SARS-CoV-2) has both direct and indirect effects on the health of people with Parkinson’s disease (PD). While direct effects comprise the clinical impact of the coronavirus disease (COVID-19) on the individual and PD symptoms [1, 2], indirect health effects [3, 4] refer to the impact of societal responses to the pandemic on people with PD, e.g., the impact of lockdowns or the delay of hospital treatments.

Evidence on the direct health effects of COVID-19 on PD is accumulating and has recently been summarized in several review articles and meta-analyses [5–13]. In general, a higher risk of PD individuals for getting infected cannot be assumed currently [5, 6, 10, 14]. Once infected with the wildtype variant, the clinical presentation of PD patients is characterized by typical COVID-19 symptoms such as fever, cough, and dyspnea, whereas some atypically present with isolated worsening of PD symptoms [6, 15]. About one-third of PD patients with COVID-19 require hospitalization [5, 8] and 12.8% [16] to 17.8% [17] of these inpatients are admitted to intensive care unit (ICU). Meta-analyses reported COVID-19 mortality rates of 12% [11], 18.9% [5] and 25.1% [8] as well as an odds ratio of 1.50 for death [10] in PD individuals, with considerable heterogeneity of study samples and settings. While figures from the community setting range from 5.7% [14] to 19.7% [18], in-hospital mortality rates are higher and range from 21.3% [19] to 35.8% [20]. Risk factors for poor COVID-19 outcomes include older age, male sex, advanced disease stage, frailty, and comorbidities such as hypertension, cardiovascular diseases, and diabetes mellitus [21–23].

As to indirect health effects, the COVID-19 pandemic disrupted health care delivery and utilization across all care settings and non-COVID-19 health conditions [3, 24] including neurological diseases [23, 25–28]. In people with PD, public health measures such as lockdowns and social distancing are associated with decreases in physical activity and quality of life and increases in anxiety and depression [1, 4, 14, 29–32]. However, for the community and the outpatient setting, survey-based data from Germany indicate that outpatient support was ensured for most PD patients [32]. In contrast, hospital stays allowing for comprehensive proactive and reactive care of PD patients often were deferred and decreased by up to 72.7% during the first pandemic wave between March and May 2020, in Germany [33].

This knowledge on the direct and indirect health effects of COVID-19 on PD is mainly based on data from early phases of the pandemic. Importantly, the second wave met a more prepared health care system as treatment options of clinical management had grown in number and user experience with the start of the second pandemic wave in Germany in early October 2020 [34]. As a possible consequence, a trend towards better outcomes has been observed in both the hospital setting [34] and the general population [35]. Despite this trend, the second wave has been described as ‘substantially stronger’ in epidemiological terms regarding absolute numbers of COVID-19 cases, deaths, ICU occupancy rates, and outbreak events (especially in nursing home facilities)[36, 37].

Regarding the public health management of the pandemic, lockdowns were initiated during both waves 2020 in Germany, with a total lockdown from March 22 on during the first wave, as well as a partial and a following total lockdown on November 2 and December 16, respectively, during the second wave [38–40]. While hospitals were called to defer non-urgent treatments and were assured financial compensation relatively early during the first wave (on March 12, 2020 [41]), these political decisions were communicated fairly later during the second wave (on November 18, 2020 [42]).

Given this background and as both clinical and public health management of COVID-19 and the pandemic, respectively, vary over time, we hypothesized that in-hospital COVID-19 outcomes in PD patients and hospital care utilization for PD may have differed between phases or waves of the pandemic. We aimed to examine two principal questions: Were there differences in outcomes and characteristics of COVID-19 inpatients with PD between the two waves? How did PD hospital care between the pandemic waves change in terms of number and characteristics of PD inpatients without COVID-19?

## Methods

### Study design

A nationwide ecological cross-sectional study was conducted to determine differences in COVID-19 outcomes of hospitalized people with PD and in hospital care utilization for PD inpatients without COVID-19 between the two pandemic waves 2020 in Germany.

### Database

We used the nationwide administrative claims database which is based on diagnosis‐related groups (G-DRG [43]; Data retrieval according to §21 KHEntgG and §24 Abs. 2 KHG; official data on file, source: Institut für das Entgeltsystem im Krankenhaus, InEK, www.g-drg.de). In Germany, all inpatient cases are encoded according to the International Classification of Diseases 10, German Modification (ICD-10-GM [44]), and the German procedure classification (operation and procedure keys, OPS [45]). Hospitals are legally required to provide comprehensive data on hospital treatment to InEK, including discharge information. ICD codes are assigned to inpatient cases with regard to the diagnoses made or confirmed by board-certified physicians during the hospital stay. Within the DRG coding system, main diagnoses (reasons for hospitalization) and secondary diagnoses (comorbidities) are shown. The database covers nearly 100% of all German hospitals (a total number of 1468 hospitals). About 20% of cases are assessed for validity by board-certified physicians of the medical service of Germany’s National Association of Statutory Health Insurance Funds, thus warranting a high quality and external validity of the data. Data were retrieved retrospectively on June 20 and November 9, 2021.

### Participants

We included all cases admitted to German hospitals during the two periods of interest which comprised March 1 to May 31 (first wave, W1, approx. covering weeks 10–22), and October 1 to December 31 (second wave, W2, approx. covering weeks 40–52) 2020 and 2019, using a previously established temporal definition of our group [46]. Based on the encoded ICD diagnoses, we formed two groups that were described regarding the outcome variables presented below. One group comprised all COVID-19 inpatients (secondary diagnosis U07.1, ‘COVID-19, virus identified’, any main diagnosis), and included subjects with PD (G20) as either main or secondary diagnosis as well as subjects without PD. The other group comprised patients admitted for PD (G20 as main diagnosis) without COVID-19 (excluding U07.1 cases). To determine the relative difference 2020 versus 2019 in numbers of hospitalizations, we analyzed all cases encoded with any main diagnosis—including those with PD (G20) as main diagnosis—and at the same time without COVID-19 (U07.1). To warrant anonymization, primary individual-level data were converted to secondary high-level data before they were retrieved.

No informed consent or ethical approval was required, as this analysis is based on anonymized secondary data that were provided by the German Federal Statistical Office and thus complied with the German data protection regulations.

### Variables

The primary outcomes were COVID-19 in-hospital mortality (discharge code ‘07’, death), ICU admission rate (OPS code ‘8-980/8-98f’, intensive care complex treatment), and the change in hospital care utilization for non-COVID-19 PD inpatients (G20, excluding U07.1), defined as the year-to-year relative change (2020 vs. 2019) in numbers of hospitalizations (i.e., main diagnoses) in relation to each period of interest (W1 and W2).

Secondary outcomes comprised demographical data including age, gender and care-dependency, PD characteristics regarding the disease stage according to Hoehn and Yahr [44] and the presence of motor fluctuations, and a number of selected comorbidities. The corresponding ICD-10-GM and OPS codes are displayed in Tables 1 and 2. The unit of analysis for frequency analyses is ‘case’. Multiple counting was avoided using key ‘06’ (discharge to another hospital). Case numbers are considered patient numbers since the number of potentially readmitted patients in the examined periods is regarded as negligible.

**Table 1:** Characteristics and outcomes of COVID-19 inpatients with and without PD

	March–May (1st wave)				October–December (2nd wave)				PD versus non-PD						2nd versus 1st wave					
	PD		Non-PD		PD		Non-PD		1st			2nd			PD			non-PD		
									RR	95% CI		RR	95% CI		RR	95% CI		RR	95% CI	
										*LL*	*UL*		*LL*	*UL*		*LL*	*UL*		*LL*	*UL*
N	775		32,858		2600		111,497													
*Demographics*																				
Age (M, SD, relative difference)	79.7	13.1	67.0	6.8	80.0	13.4	66.7	7.3	0.160*	n.a	n.a	**0.167**^†^	n.a	n.a	0.003^‡^	n.a	n.a	**− 0.004**^§^	n.a	n.a
Female gender	306	39.5%	14,955	45.5%	1090	41.9%	55,015	49.3%	**0.868**	0.794	0.947	**0.850**	0.812	0.889	1.062	0.962	1.171	**1.084**	1.070	1.099
Care dependency (OPS 9-984.6/7/8/9/a)	582	75.1%	9293	28.3%	2096	80.6%	37,311	33.5%	**2.655**	2.541	2.775	**2.409**	2.360	2.459	**1.073**	1.027	1.123	**1.183**	1.161	1.206
*PD characteristics*																				
HY < 3 (G20.0-)	89	11.5%	n.a	n.a	327	12.5%	n.a	n.a	n.a	n.a	n.a	n.a	n.a	n.a	1.095	0.879	1.365	n.a	n.a	n.a
HY 3–4 (G20.1-)	230	29.7%	n.a	n.a	661	25.7%	n.a	n.a	n.a	n.a	n.a	n.a	n.a	n.a	**0.857**	0.755	0.972	n.a	n.a	n.a
HY 5 (G20.2-)	55	7.1%	n.a	n.a	149	6.0%	n.a	n.a	n.a	n.a	n.a	n.a	n.a	n.a	0.808	0.599	1.089	n.a	n.a	n.a
HY n.s. (G20.9-)	401	51.7%	n.a	n.a	1463	55.9%	n.a	n.a	n.a	n.a	n.a	n.a	n.a	n.a	**1.087**	1.008	1.173	n.a	n.a	n.a
Motor fluctuations (G20.-1)	104	13.4%	n.a	n.a	272	10.9%	n.a	n.a	n.a	n.a	n.a	n.a	n.a	n.a	**0.780**	0.631	0.963	n.a	n.a	n.a
*Comorbidities*																				
Diabetes mellitus, type 2 (E11)	196	25.3%	7455	22.7%	730	28.1%	26,792	24.0%	1.115	0.986	1.260	**1.168**	1.098	1.244	1.110	0.969	1.272	**1.059**	1.036	1.083
Obesity (E66)	21	2.7%	1693	5.2%	53	2.0%	5600	5.0%	**0.526**	0.344	0.804	**0.406**	0.311	0.530	0.752	0.457	1.239	0.975	0.925	1.028
Hypertension (I10)	414	53.4%	15,286	46.5%	1530	58.8%	49,762	44.6%	**1.148**	1.074	1.228	**1.319**	1.276	1.362	**1.102**	1.024	1.185	**0.959**	0.947	0.972
Chronic ischaemic heart disease (I25)	163	21.0%	5673	17.3%	535	20.6%	19,007	17.0%	**1.218**	1.061	1.399	**1.207**	1.118	1.303	0.978	0.837	1.143	0.987	0.961	1.014
Cerebrovascular disease (I69)	40	5.2%	1083	3.3%	140	5.4%	3451	3.1%	**1.566**	1.151	2.130	**1.740**	1.476	2.051	1.043	0.741	1.469	0.939	0.878	1.004
COPD (J44)	47	6.1%	2218	6.8%	150	5.8%	6780	6.1%	0.898	0.679	1.189	0.949	0.811	1.110	0.951	0.692	1.307	**0.901**	0.860	0.944
Chronic kidney disease (N18)	170	21.9%	5703	17.4%	649	25.0%	19,512	17.5%	**1.264**	1.104	1.446	**1.426**	1.333	1.527	1.138	0.981	1.320	1.008	0.982	1.036
*Outcomes*																				
In-hospital mortality (discharge code 07)	253	32.6%	6605	20.1%	963	37.0%	21,565	19.3%	**1.624**	1.464	1.801	**1.915**	1.819	2.016	**1.135**	1.013	1.270	**0.962**	0.939	0.986
ICU treatment (OPS 8-980/8-98f)	168	21.7%	9845	30.0%	455	17.5%	22,685	20.3%	**0.723**	0.632	0.828	**0.860**	0.791	0.936	**0.807**	0.690	0.945	**0.679**	0.665	0.693

*HY* Hoehn and Yahr stage, *n.s*. not specified, *n.a.* not applicable, significant results (*p* < 0.05) are marked in bold

*p < 0.0001, t(33,631) = 49.9; ^†^*p* < 0.0001, t(114,095) = 89.5; ^‡^*p* = 0.583, t(3373) = 0.55; ^§^*p* < 0.0001, t(144,353) = 6.65

**Table 2:** Characteristics and outcomes of people admitted for PD without COVID-19

	March–May (1st wave)				October–December (2nd wave)				2nd versus 1st (2020)			2020 versus 2019 (1st)			2020 versus 2019 (2nd)		
	2020		2019		2020		2019		RR	95% CI		RR	95% CI		RR	95% CI	
										*LL*	*UL*		*LL*	*UL*		*LL*	*UL*
N	6618		11,820		7802		10,129		n.a	n.a	n.a	n.a	n.a	n.a	n.a	n.a	n.a
*Demographics*																	
Age (M, SD, relative difference)	73.8	8.4	73.9	8.4	73.3	8.2	73.9	8.4	− 0.006*	n.a	n.a	−0.002^†^	n.a	n.a	− **0.007**^‡^	n.a	n.a
Female gender	2497	37.7%	4727	40.0%	3104	39.8%	3971	39.2%	**1.054**	1.012	1.099	**0.943**	0.908	0.980	1.015	0.978	1.053
Care dependency (OPS 9-984.6/7/8/9/a)	3918	59.2%	6527	55.2%	4470	57.3%	5537	54.7%	**0.968**	0.941	0.995	**1.072**	1.045	1.100	**1.048**	1.021	1.076
*PD characteristics*																	
HY < 3 (G20.0)	785	11.9%	1434	12.1%	933	12.0%	1356	13.4%	1.008	0.922	1.102	0.978	0.901	1.061	**0.893**	0.826	0.966
HY 3–4 (G20.1)	4381	66.2%	7923	67.0%	5320	68.2%	6782	67.0%	**1.030**	1.007	1.054	0.988	0.967	1.009	1.018	0.998	1.039
HY 5 (G20.2)	838	12.7%	1381	11.7%	824	10.6%	1071	10.6%	**0.834**	0.762	0.913	**1.084**	1.000	1.174	0.999	0.917	1.088
HY n.s. (G20.9)	614	9.3%	1082	9.2%	725	9.3%	920	9.1%	1.002	0.904	1.110	1.014	0.922	1.114	1.023	0.932	1.123
Motor fluctuations (G20.-1)	4159	62.8%	7176	60.7%	4671	59.9%	6254	61.7%	**0.953**	0.928	0.978	**1.035**	1.011	1.060	**0.970**	0.947	0.993
*Comorbidities*																	
Diabetes mellitus, type 2 (E11)	1072	16.2%	1953	16.5%	1168	15.0%	1608	15.9%	**0.924**	0.856	0.997	0.980	0.916	1.049	0.943	0.880	1.011
Obesity (E66)	175	2.6%	383	3.2%	214	2.7%	310	3.1%	1.037	0.852	1.263	**0.816**	0.684	0.973	0.896	0.755	1.064
Hypertension (I10)	3290	49.7%	5762	48.7%	3623	46.4%	4899	48.4%	**0.934**	0.903	0.966	1.020	0.989	1.051	**0.960**	0.931	0.991
Chronic ischaemic heart disease (I25)	822	12.4%	1403	11.9%	812	10.4%	1174	11.6%	**0.838**	0.765	0.918	1.046	0.965	1.134	**0.898**	0.825	0.977
Cerebrovascular disease (I69)	200	3.0%	342	2.9%	212	2.7%	261	2.6%	0.899	0.743	1.088	1.044	0.880	1.240	1.055	0.882	1.261
COPD (J44)	179	2.7%	338	2.9%	171	2.2%	275	2.7%	**0.810**	0.659	0.997	0.946	0.791	1.131	**0.807**	0.669	0.975
Chronic kidney disease (N18)	698	10.5%	1215	10.3%	832	10.7%	1040	10.3%	1.011	0.919	1.112	1.026	0.940	1.121	1.039	0.953	1.132
*Outcomes*																	
In-hospital mortality (07)	117	1.8%	135	1.1%	82	1.1%	130	1.3%	**0.594**	0.449	0.787	**1.548**	1.211	1.979	0.819	0.622	1.078
ICU treatment (OPS 8-980/8-98f)	236	3.6%	302	2.6%	215	2.8%	227	2.2%	**0.773**	0.644	0.927	**1.396**	1.180	1.650	**1.230**	1.023	1.478

*HY* Hoehn and Yahr stage, *n.s.* not specified, *n.a.* not applicable, significant results (*p* < 0.05) are marked in bold

*p = 0.0003, t(14,418) = 3.61; ^†^*p* = 0.438, t(18,436) = 0.78; ^‡^*p* < 0.0001, t(17,929) = 4.79

Primary and secondary outcomes were described separately for the two inpatient groups, i.e., the COVID-19 and the non-COVID-19 group.

### Statistical methods

Categorical variables were reported as absolute and relative frequencies, whereas continuous variables were reported as mean and standard deviation. Univariate analysis for continuous variables was conducted with student’s t-test. To describe differences in categorical variables between groups and subgroups, we calculated risks ratios (RR; also referred to as relative risks) with 95% confidence intervals (CI) under a random-effects model. Student’s t-test was performed using the online t test calculator from GraphPad (https://www.graphpad.com/quickcalcs/ttest1, GraphPad Software Inc., San Diego, CA, USA). RR calculations were conducted with the Stata Statistical Software Release 17 for Mac (StataCorp LLC, College Station, TX, USA).

This study followed the STROBE reporting guidelines (Strengthening the Reporting of Observational Studies in Epidemiology [47]).

## Results

### Inpatients with COVID-19

While ICU admission rates of COVID-19 inpatients decreased in both the PD and non-PD group during the second wave, compared to the first, in-hospital mortality increased between waves in PD subjects only (Table 1). During the second wave, care-dependency was more frequent in COVID-19 inpatients both with and without PD (Table 1). For PD subjects, no significant changes in age and gender proportion were observed for the second wave. HY stages 3–4 occurred significantly less frequently than during the first wave, whereas undefined disease stages were significantly more frequent. In trend, HY stages < 3 were more frequent, whereas HY stages 5 were less frequent. During the second wave, COVID-19 inpatients with PD were less frequently affected by motor fluctuations. As to comorbidities, the frequency of hypertension increased between waves in PD (Table 1).

**Fig. 1:**
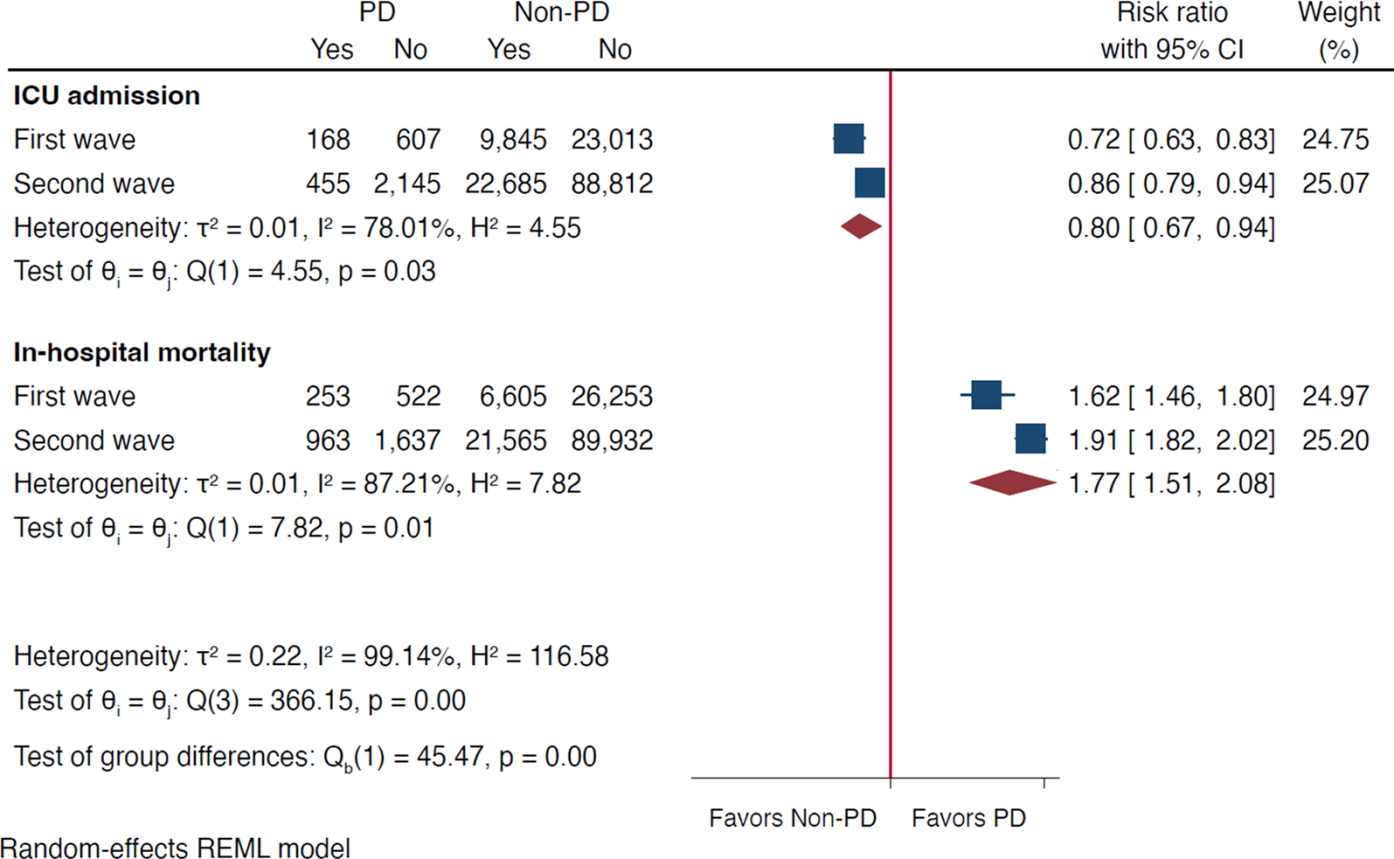
COVID-19 outcomes in PD and non-PD subjects

Compared to non-PD subjects, during both waves, COVID-19 inpatients with PD showed higher in-hospital mortality (pooled RR: 1.77, 95% CI: 1.51–2.08; Fig. 1), lower ICU admission rates (pooled RR: 0.80, 95% CI: 0.67–0.94), more frequent long-term care-dependency, and were more frequently male and older (Table 1). They more often suffered from type 2 diabetes mellitus (applies to the second wave only), arterial hypertension, cardio- and cerebrovascular disease, and chronic kidney disease, and less often from obesity (Table 1).

### Inpatients without COVID-19

**Fig. 2:**
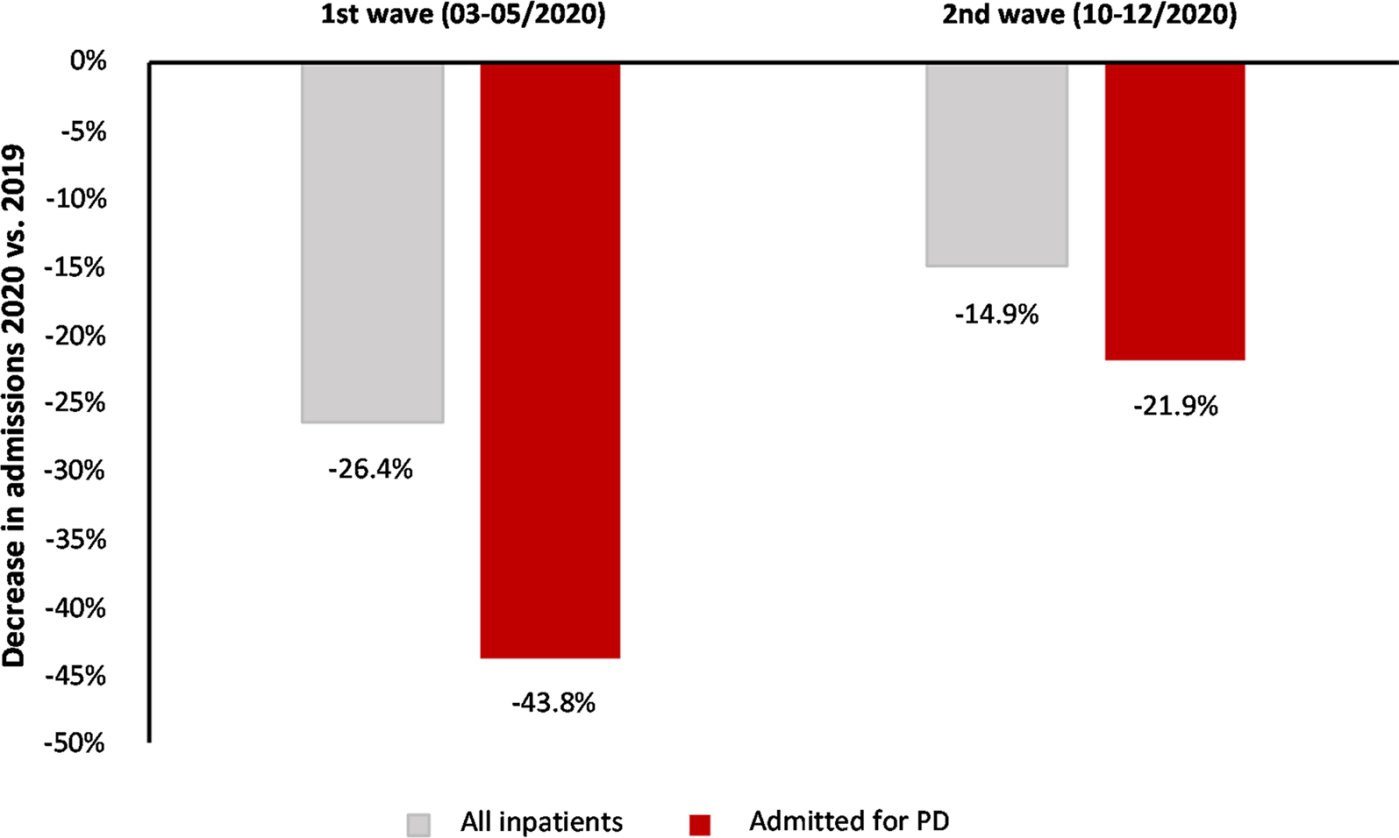
Decrease in admissions 2020 vs. 2019 for any and Parkinson’s disease

In 2020, overall admissions decreased by 26.4% and 14.9% during the first and the second wave, respectively (Fig. 2, Additional file 1), compared to 2019. PD admissions more markedly decreased by 43.8% and 21.9%, respectively. Overall, the decrease in hospital care utilization for non-COVID-19 conditions was stronger for PD than for overall admissions during both waves, and smaller during the second wave.

Compared to the first wave, PD inpatients without COVID-19 showed lower in-hospital mortality and ICU admission rates during the second wave (Table 2) and were slightly younger, more frequently female, and less frequently long-term care-dependent (Table 2). HY stages 5 were less frequent, whereas HY stages 3–4 and < 3 occurred more frequently.

During both waves in 2020, people admitted for PD (without COVID-19) were more frequently treated in ICU than people admitted for PD during the corresponding pre-pandemic periods in 2019 (Table 2). Likewise, care-dependency was more frequent in inpatients during both waves, 2020. During the first wave, PD inpatients were more frequently male, more frequently allocated to HY stage 5, and in trend showed more comorbidities such as hypertension, chronic kidney disease, and cardio- and cerebrovascular diseases, compared to 2019. In addition, PD inpatients showed higher in-hospital mortality during the first wave than during the same episode in the pre-pandemic year 2019, as reported previously [23]. In contrast, during the second wave, PD inpatients showed no increased in-hospital mortality and fewer comorbidities and were younger, compared to 2019 (Table 2).

## Discussion

This was a nationwide cross-sectional study to determine the differences in COVID-19 outcomes of hospitalized people with PD and in hospital care utilization for PD inpatients without COVID-19 between the two pandemic waves 2020 in Germany.

### COVID-19 outcomes

We showed that people with PD hospitalized with COVID-19 during the second wave in Germany exhibited higher in-hospital mortality (37.0%) along with lower ICU admission rates (17.5%) than during the first wave, and compared to COVID-19 inpatients without PD.

In-hospital mortality worsened in the PD group over time, whereas it did not change in published average data [34], and improved in the non-PD group. The increased mortality could be related to the characteristics of the PD inpatient group during the second wave, i.e., increased frequency of long-term care-dependency compared to the first wave, older age in trend, and increased frequency of hypertension as a comorbidity with substantial contribution to poor COVID-19 outcomes [18, 48, 49].

These characteristics are likely to have been influenced by patterns of viral spread during the second wave. While the partial lockdown at the beginning of November did not curb the growth in COVID-19 incidences among people aged > 60 years [36, 39], only with the total lockdown in mid-December incidence rates decreased across all age groups [39]. Long-term care facility outbreaks were more frequent per week during the second wave [36], larger, showed more cases in the elderly and females and were less effectively affected by non-pharmaceutical interventions (NPIs) such as lockdowns [37]. Outbreak characteristics might thus have been mirrored by PD study population characteristics, i.e., not significantly and significantly increased proportions of females and long-term care-dependent individuals, respectively. Therefore, risk factors of mortality could have been reinforced by the epidemiological characteristics of the second wave.

The prognosis might further have been affected unfavorably by less or suboptimal treatment of PD patients. In more detail, lower ICU admission rates of COVID-19 inpatients with PD during the second wave do not necessarily indicate an improvement in outcomes resulting from improved efficacy of non-invasive treatments [34]. Less treatment could rather have resulted from a stronger tendency toward palliative approaches in advanced PD, as suggested previously [50]. Reduced ICU admission rates may additionally reflect suboptimal in-hospital care as a possible result of atypical COVID-19 clinical presentation in PD patients, e.g., alterations of mental or neurological state [48, 51] rather than shortness of breath [14, 52, 53], which may be particularly true for additionally demented individuals [22]. Together with a more considerable workload—ICU occupancy rates were higher during the second wave [54]—and consecutively reduced resources, unconsciously reduced attention to atypical or complex cases might have further contributed to the reduced ICU treatment and increased mortality of PD inpatients.

Of note, in-hospital mortality was higher in PD inpatients compared to non-PD inpatients during both waves, although this study was not designed to assess adjusted risks of COVID-19 mortality in PD. This difference is most likely related to older age, male preponderance, more frequent long-term care-dependency, and risk comorbidities in the PD group. These factors have been shown to increase the risk of high COVID-19 mortality in PD [21–23]. Whereas some studies suggest an increased COVID-19 mortality risk for PD [19, 23] or neurodegenerative diseases [50] independent of age [19, 23, 50] and sex [19, 50], further evidence does not support conclusions on PD as an independent risk factor for COVID-19 mortality (e.g., [22]), and recent reviews are backing this notion [5–13].

Overall, the figures of COVID-19-associated in-hospital mortality are higher yet comparable to those found in previous cohort [22, 48] and cross-sectional [20, 55] studies, with in-hospital mortality rates ranging from 32.0% [48] to 35.8% [20] in PD samples of roughly the same age, and excluding figures from smaller (n < 25) studies [56] and case series [2, 15, 51, 57]. However, a large case–control study revealed a lower mortality rate of 21.3% [19], which could be due to the matching of that sample to demographical data leading to assimilation to the lower figures reported in the community [14, 16, 18]. In general, a selection bias may have skewed mortality figures, as COVID-19 hospital care may have been utilized by the most severely affected only, given stronger fears and anxiety in PD individuals than in the general population [30, 58] and cautious stay-at-home attitudes.

Taken together, in contrast to a trend towards better outcomes in average COVID-19 inpatients [34] and the general population [35] during the second wave that met a more experienced health care system, outcomes became poorer for COVID-19 inpatients with PD. This may be associated with clinical and demographical characteristics (e.g., atypical clinical presentation, more hypertension, and long-term care-dependency), epidemiological factors (e.g., larger outbreak events in long-term care facilities, possibly due to ineffective partial lockdown), and aspects of clinical management (e.g., less treatment related to a stronger tendency toward palliative approaches in advanced PD, or reduced health care resources).

### Hospital care utilization

Hospital care utilization for non-COVID-19 conditions was decreased in 2020 for overall admissions and with pronounced decreases for PD. Reasons for decreases in admissions include public health measures such as deferral of elective hospital stays and lockdowns as well as common behaviors such as social distancing involving stay-at-home attitudes, or fears of contracting SARS-CoV-2 at the hospital [3, 24]. One possible reason for stronger reductions in PD admissions may be subjective issues like worries and fears associated with COVID-19 [30, 59] since anxiety is more common in PD than in the general population [58]. Another reason could be cautious attitudes in people counseling PD patients, e.g., caregivers, therapists, or doctors. Most importantly, compared to people needing urgent treatments, e.g., oncologic or emergency patients, people with chronic conditions like PD may not have been prioritized for hospitalization.

Crucially, common reasons for PD admission [60–62] like delirium, infections, disease exacerbations, or falls can be assumed not to decrease substantially during pandemic events, not to mention the necessity of proactive hospital stays for careful dopaminergic titration, intensive multidisciplinary rehabilitation, or management of device-aided therapies [63]. Substantial reductions in hospital care utilization are therefore contrasted by a substantial need for hospital care in PD patients. This continuous need together with a certain effect of habituation to pandemic conditions may be one reason that decreases in PD hospital care utilization were less marked during the second wave. Further, differences between waves in hospital care utilization may be related to differences in public health measures and common behavior. Specifically, during the second wave, obligations to defer non-urgent treatments and concessions of financial recompensating for hospitals were politically communicated later, i.e., seven [42] instead of two [41] weeks after the start of the respective wave. In addition, cell phone mobility data indicate less social distancing during the second wave [64] which might have been accompanied by fewer fears and caution, and lockdowns that were imposed relatively late during the second wave.

Interestingly, PD patients without COVID-19 were more likely to have poor in-hospital outcomes in 2020 than in 2019. For the overall group of neurological inpatients, a higher relative in-hospital mortality risk adjusted for age, sex, and comorbidities has been shown in a study from 87 German hospitals [27]. This was albeit not significant when COVID-19 patients were excluded. We suggest that poor outcomes in the first pandemic year are explained by a selection bias where only the people most in need utilized hospital care. Indeed, care-dependency was more frequent in 2020 PD inpatients, as our data show. Whereas this selection effect might have been more pronounced during the first wave with PD inpatients being more advanced, showing more comorbidities and higher in-hospital mortality, it was less prominent during the second wave with less effective public containment strategies and younger, less advanced PD inpatients with fewer comorbidities. As stated above, reduced hospital care resources and distraction of attention by the pandemic could have facilitated poor outcomes of in-hospital complications frequently occurring in PD such as infections, confusion, postural hypotension, and falls [65, 66]. Therefore, non-COVID-19 in-hospital outcomes in 2020 may be related to both objective (e.g., advanced disease stage, comorbidities) and subjective matters (e.g., reduced fears and habituation).

### Limitations and strengths

Several limitations of this study have to be considered when interpreting the results. As the data provided were aggregated to a high-level scale, a comprehensive adjustment for confounders such as age, sex, or disease stage could not be performed. In addition, the period of study comprised only two-thirds of the second wave, as it continued – with decreasing COVID-19 incidence rates – in the first eight weeks of 2021. However, this methodology made the findings more comparable to previous analyses. Another limitation is a selection bias, which has been discussed above and concerns the results related to mortality rates and characteristics of COVID-19 patients. Notably, no information on COVID-19 patients with PD in the outpatient sector or community was provided. Moreover, even if the quality and validity of the used G-DRG database are ensured by regular testing, coding errors in times of tight resources cannot be ruled out. Additionally, in-hospital mortality, strictly speaking, cannot be completely attributed to COVID-19 (dying “of” COVID-19). However, an autopsy study [67] identified COVID-19 as an underlying cause of death in 86% of cases. Of note, during both waves, the wild-type variant of SARS-CoV-2 was predominant and a comprehensive vaccination program was still absent. This limits the generalizability of our findings to following pandemic waves or phases.

To the best of our knowledge, this paper is the first attempt to describe temporal trends in direct and indirect effects of the COVID-19 pandemic on PD patients throughout its course. The findings which are based on nationwide data may inform management of future COVID-19 outbreaks or pandemics.

In general, PD care during following pandemic waves may be improved by transforming the care system toward an integrated network approach [68] with enhanced interprofessional communication [69] and the use of telemedicine [70], while maintaining an attitude of preparedness [71].

## Conclusions

In-hospital COVID-19 outcomes and hospital care utilization of PD patients differed between the two pandemic waves 2020 in Germany. Direct and indirect effects of the COVID-19 pandemic on health of people living with PD do not only vary in space (as shown by differential mortality figures across care settings and countries) but also in time and together with changes in clinical and public health management, knowledge, and attitudes as well as epidemiological characteristics of the pandemic. Whereas data on in-hospital COVID-19 outcomes of PD patients during the second pandemic wave indicate a deterioration, data on hospital care utilization suggest a favorable yet incomplete recovery of PD hospital care during the second wave.

## Supplementary Information

**Additional file 1: Table. **Decrease in admissions 2020 versus 2019 for any and Parkinson’s disease during the first and second pandemic wave in Germany, 2020.

## Data Availability

The data that support the findings of this study are available from the corresponding author upon reasonable request.

## Abbreviations

SARS-CoV-2: Severe acute respiratory coronavirus 2
PD: Parkinson’s disease
COVID-19: Coronavirus disease
G-DRG: German diagnosis-related groups
ICU: Intensive care unit
ICD-10-GM: International Classification of Diseases 10, German Modification
OPS: German procedure classification, operation and procedure keys
NPI: Non-pharmaceutical intervention
