# Supplementary material for: COVID-19 outcomes in hospitalized Parkinson’s disease patients in two pandemic waves in 2020: a nationwide cross-sectional study from Germany
Source: Neurol Res Pract. 2022 Jul 11;4:27. doi: 10.1186/s42466-022-00192-x (PMC9271552; doi:10.1186/s42466-022-00192-x)
Supplement: Supplementary file 1 — Additional file 1: Table. Decrease in admissions 2020 versus 2019 for any and Parkinson’s disease during the first and second pandemic wave in Germany, 2020. [file 42466_2022_192_MOESM1_ESM.pdf]

Table: Decrease in admissions 2020 vs. 2019 for any and Parkinson's disease during the first and second pandemic wave in Germany, 2020

	All inpatients		Inpatients admitted for PD (without COVID-19)	
	March-May	October-December	March-May	October-December
2020, n	3,480,354	3,695,695	6,644	7,912
2019, n	4,730,927	4,345,170	11,820	10,129
Difference, n	-1,250,573	-649,475	-5176	-2217
Difference, %	-26.4%	-14.9%	-43.8%	-21.9%
